# Construction of Triphenylamine-Based Aggregation-Induced Emission Luminogens for Lysosomes Imaging and Its Application in the Photodynamic Therapy of Cancer Cells

**DOI:** 10.3390/molecules30112272

**Published:** 2025-05-22

**Authors:** Zhanguo Sun, Bin Liu, Huijun Liu

**Affiliations:** 1 School of Chemistry and Chemical Engineering, Shanxi Datong University, Datong 037009, China; dtdxlhj@163.com; 2 Key Laboratory of Chemical Biology and Molecular Engineering, Ministry of Education, Institute of Molecular Science, Shanxi University, Taiyuan 030006, China; liubin@sxu.edu.cn

**Keywords:** lysosomes, bright yellow emission, AIEgens, photodynamic therapy

## Abstract

Lysosomes are important acidic subcellular organelles whose dysfunction can lead to some related diseases. The development of new lysosome-imaging-guided AIEgens for the photodynamic therapy of cancer cells is important. In this work, two novel organic compounds with AIE characteristics, namely, TPAB-CF_3_ and TPAB-diCF_3_, were designed and synthesized by introducing the weakly basic morpholinyl moiety with lysosome-targeting ability into a triphenylamine-based luminogen. The distorted spatial feature of TPA and the D_1_-D_2_-π-A structure of these AIEgens prevented the aggregation-caused quenching of traditional fluorescent molecules and efficiently promoted the separation of the HOMO and LUMO. The outcomes were AIE features and a narrow single-triplet energy gap. Furthermore, TPAB-CF_3_ and TPAB-diCF_3_ showed bright yellow fluorescence emission peaks at 577 and 601 nm; large Stokes shifts of 234 and 256 nm, respectively; and excellent lysosome-targeted imaging of HeLa cells (Pearson’s coefficient = 0.90). In addition to the good ^1^O_2_-generation ability under light irradiation, these AIEgens achieved the high-efficiency bright lysosome imaging-guided photodynamic killing of cancer cells under white-light irradiation.

## 1. Introduction

Lysosomes are ubiquitous membrane-bound subcellular organelles in eukaryotic cells, which contain an acidic interior and up to 60 kinds of hydrolases [1]. They are the central sites for the degradation and recycling of macromolecules [2,3]. Numerous studies have shown that they are key participants in intracellular homeostasis and play important roles in various physiological functions, such as plasma membrane repair, cholesterol homeostasis, cell signal transduction, and cell death [4,5,6,7]. Lysosomal disorders have profound effects on homeostasis in the body and can lead to lysosomal storage disorders, neurological disorders, cardiovascular diseases, and even cancer [8,9]. Rapidly dividing cancer cells highly rely on the function of lysosomes. During transformation and cancer progression, the volume, composition, or localization of lysosomes have significant alters. Lysosomal cathepsins increase and mislocalize during tumor development, leading to their secretion in abnormal forms [10]. Abnormal secreted cathepsins may stimulate angiogenesis and promote tumor growth and aggression [11]. Therefore, the effective monitoring of lysosomes within cells and the photodynamic therapy (PDT) targeting lysosomes for cancer cells has great significance for killing cancer cells [12].

As an emerging technology for cancer cell elimination, PDT has the advantage of effectiveness regardless of drug resistance and traumatic injury [13,14]. The effectiveness of PDT on cancer cell elimination primarily depends on the photosensitizer (PS). It is irradiated with light and generates reactive oxygen species (ROS) like singlet oxygen (^1^O_2_), which can lead to the death of cancer cells [15]. Since the first PDT agent hematoporphyrin was applied in a clinic in 1955, a large number of PSs including porphyrin, BODIPY, cyanin, and perylene derivatives have been exploited as lysosome-targeted imaging fluorescent dyes [16,17,18,19,20]. However, most traditional PSs suffer from the aggregation-caused quenching (ACQ) effect. It is not conducive to observe the performance of image-guided PDT in aqueous environments [21,22].

To overcome the weakness of conventional fluorescent dyes with the ACQ effect, a new concept named aggregation-induced emission (AIE) luminogens (AIEgens) was coined in 2001 by Tang’s research group [23]. Molecules with AIE features exhibit bright emissions in an aggregation state due to the intramolecular motion restriction, which is conducive to application in the field of biological imaging [24]. After more than 20 years of development, multiple functionalized AIEgens with specific subcellular organelle targeting (lipid droplet and mitochondria or lysosome) and efficient ^1^O_2_ production have been reportedly applied in PDT [25,26,27,28,29,30,31]. Considering the significant role of lysosomes in maintaining the normal metabolic balance of cells, although some novel AIEgens have been reported, the construction of AIEgens with lysosome-targeted imaging and PDT ability is still in demand.

As an effective strategy, extending π-conjugated chains to the AIE skeleton and increasing the push–pull effect is suitable for designing AIEgens with a D_1_-D_2_-π-A structure [32,33]. Such AIEgens with a strong push–pull effect more easily separate the HOMO and LUMO. The outcome is a smaller ΔE_S1−T1_, which possesses compound effective ^1^O_2_ production [34,35]. Hence, by modifying the target group (e.g., the weakly basic morpholine group) to triphenylamine-based AIEgens, the compounds can be endowed with excellent lysosome-targeting performance and effective ^1^O_2_ generation for the PDT effect.

**Scheme 1:**
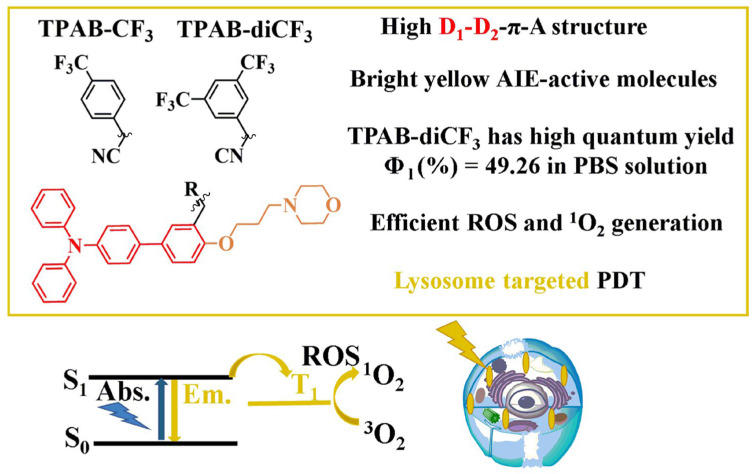
Features and PDT application diagram of TPA-based lysosome-targeted AIEgens.

In the present study, two AIEgens (TPAB-CF_3_ and TPAB-diCF_3_) were designed and synthesized by grafting morpholinyl moiety with triphenylamine-based luminogen (Scheme 1). These compounds possess good biocompatibility and suitable *pk*_a_ values. Both AIEgens can target and image lysosomes in living cells with bright yellow emissions and high specificity. With the aid of the D_1_-D_2_-π-A structure of TPAB-CF_3_ and TPAB-diCF_3_, they can effectively generate ROS, especially ^1^O_2_, thereby enabling the ablation of cancer cells under light irradiation through the PDT effect.

## 2. Results and Discussion

### 2.1. Synthesis and Structural Analysis of Compounds TPAB-CF_3_ and TPAB-diCF_3_

**Scheme 2:**
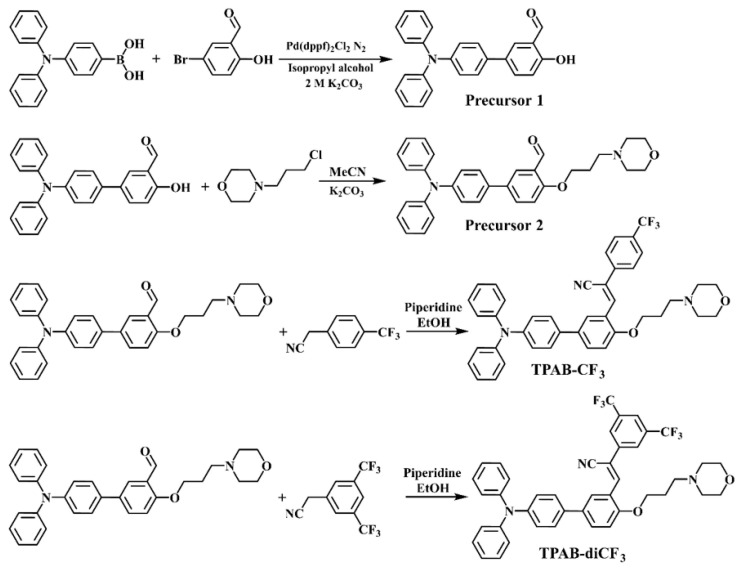
Synthesis routes of TPAB-CF_3_ and TPAB-diCF_3_.

As depicted in Scheme 2, two novel AIEgens TPAB-CF_3_ and TPAB-diCF_3_ were designed and synthesized by simple steps. First, under the catalysis of Pd(dppf)_2_Cl_2_ and N_2_ protection, 4-(diphenylamino)-phenylboronic acid and 5-bromosalicylaldehyde were refluxed in a mixture of isopropyl alcohol and potassium carbonate solution for 12 h to obtain precursor 1 by Suzuki coupling reaction. Second, 4-(3-chloropropyl)morpholine and precursor 1 were refluxed in an acetonitrile solution for 4 h. This step introduced the morpholine group with weak alkalinity into the hydroxyl site of precursor 1, thereby producing precursor 2. Finally, through the catalytic action of piperidine, 4-(trifluoromethyl)phenylacetonitrile or 3,5-bis(trifluoromethyl)phenylacetonitrile was reacted with precursor 2 in an anhydrous ethanol solution for 4 h by Knoevenagel condensation. The product was purified to obtain the target compounds TPAB-CF_3_ and TPAB-diCF_3_, respectively. These compounds were characterized by ^1^H NMR and high-resolution mass spectrometry (Figures S1–S6).

All these molecules had typical D-A structural characteristics formed with a strong electron-donating group TPA (D_1_) and electron-withdrawing group (A) via a weak electron-rich benzene ring (D_2_) bridge. Owing to the strong electron-donating property of TPA and suitable electron-withdrawing ability of the cyano (-CN) and trifluoromethyl (-CF_3_) groups, as well as the extended π-conjugated chain linkage of these molecules, they exhibited a pronounced push–pull effect. This effect was expected to contribute to the formation of narrow-bandgap compounds and thus facilitate their effective ROS-generation ability under light irradiation. Furthermore, owing to the distorted structure of TPA and the weakly basic morpholine group of these compounds, they can be easily enriched with acidic lysosomes and exhibit bright emission luminescence with AIE properties.

Given the structural design features of these compounds, they are bound to possess narrow bandgaps, bright fluorescence, and effective ROS generation for lysosome-targeting AIE-type molecules with multifunctional properties.

### 2.2. Spectral Properties of Compounds TPAB-CF_3_ and TPAB-diCF_3_

**Figure 1:**
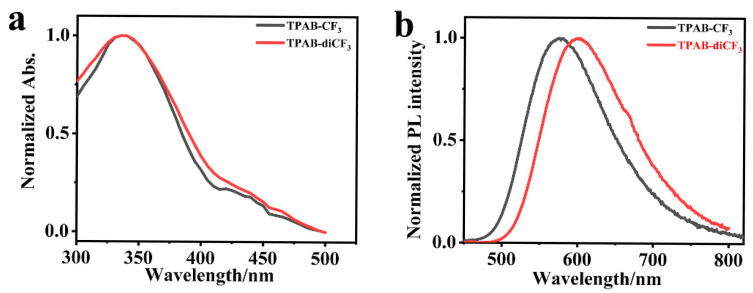
(**a**) Normalized absorption spectra of TPAB-CF_3_ and TPAB-diCF_3_; (**b**) Normalized PL spectra of TPAB-CF_3_ (λ_ex_ = 343 nm) and TPAB-diCF_3_ (λ_ex_ = 345 nm) in PBS buffer solution. Concentration: 10 μM. PBS buffer solution: pH = 7.40.

The UV–vis and PL spectra of TPAB-CF_3_ and TPAB-diCF_3_ in PBS (Phosphate Buffered Saline) are shown in Figure 1a. They display maximum absorption at 343 and 345 nm, respectively. Compared with the molecular structure of TPAB-CF_3_ and TPAB-diCF_3_, they had electron-donating unit TPA (D_1_) and phenyl group (D_2_), with the latter having a stronger ability than the electron-withdrawing group (diCF_3_ > CF_3_). Therefore, the latter had a slightly longer absorption wavelength. Due to the strong D-A structure of the compounds, their maximum emission wavelengths in PBS peaked at 577 and 601 nm. Consequently, the Stokes shift of these compounds reached 234 and 256 nm. Compared with red/near-infrared-emitting fluorophores, although these compounds exhibited yellow fluorescence emissions, their large Stokes shift still enabled them to have potential applications in biological imaging [36].

**Figure 2:**
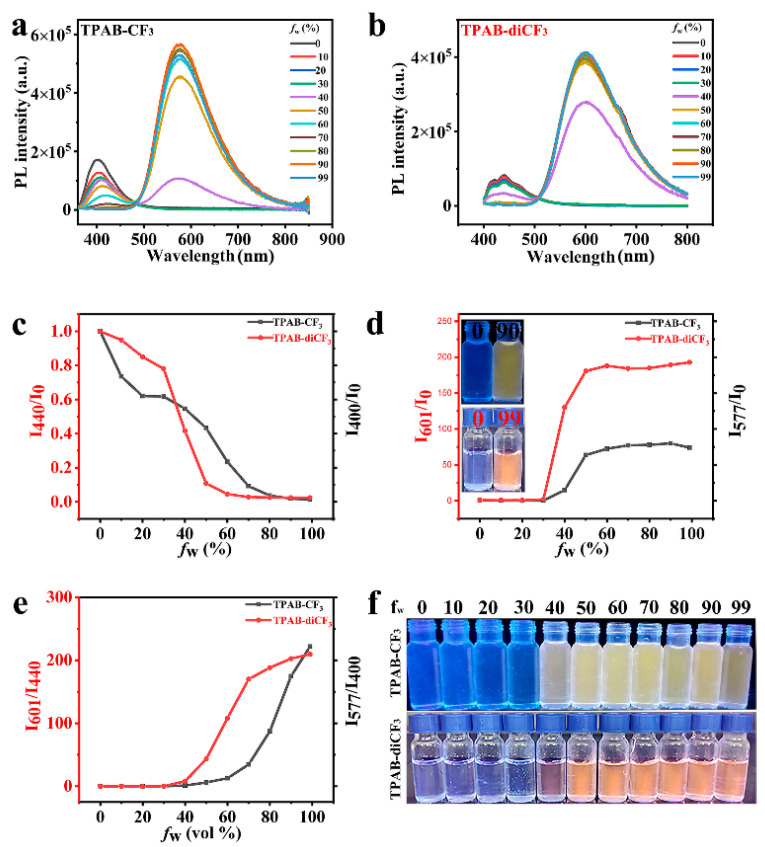
(**a**) PL spectra of TPAB-CF_3_ (**b**) and TPAB-diCF_3_ in DMSO/H_2_O system with different *f*_w_ values. (**c**) Relative fluorescence intensities of TPAB-CF_3_ and TPAB-diCF_3_ at 400 and 440 nm (**d**) and at 577 and 601 nm in DMSO/H_2_O system with different *f*_w_ values; inset photo: Photos of TPAB-CF_3_ and TPAB-diCF_3_ under a 365 nm ultraviolet lamp. (**e**) Relative fluorescence intensities of TPAB-CF_3_ (I_577_/I_400_) and TPAB-diCF_3_ (I_601_/I_440_) in DMSO/H_2_O system with different *f*_w_ values. (**f**) Photo of TPAB-CF_3_ and TPAB-diCF_3_ under 365 nm ultraviolet light in DMSO/H_2_O system with different *f*_w_ values. The concentration of compounds is 10 μM.

Based on the fact that TPA is a famous module with an electron-donating feature and a distorted structure, it is often used for constructing AIE molecules [36]. The AIE properties of TPAB-CF_3_ and TPAB-diCF_3_ were investigated in a DMSO/H_2_O system (Figure 2). As shown in Figure 2a,b, in pure DMSO, TPAB-CF_3_ and TPAB-diCF_3_ exhibited moderate intensity fluorescence emissions at 400 and 440 nm, respectively. With an increased water fraction (*f*_w_/%) from 0 to 99, the fluorescence intensity of these compounds continuously decreased at 400 and 440 nm, even completely quenching (Figure 2c). Meanwhile, Figure 2d shows that TPAB-CF_3_ and TPAB-diCF_3_ had new fluorescence emission peaks at 577 and 601 nm, respectively. With increased *f*_w_ from 0 to 30, the relatively larger fraction of DMSO in the mixture solution conferred these compounds with good solubility in it. In addition to the twisted intramolecular charge transfer (TICT) property of the compounds’ D-A structure, their fluorescence emission intensity slightly decreased [36]. However, when *f*_w_ continued to increase from 30 to 50, the poor solvent significantly increased. The aggregation effect of the compounds dramatically increased, rapidly exceeding the TICT effect. The outcomes were that the nonradiative transition of compounds weakened and the radiative transition of compounds enhanced [37], leading to sharply increased fluorescence intensity. As *f*_w_ continued to increase, the fluorescence intensity of the compounds still gradually increased. When *f*_w_ reached 90 and 99, the compounds displayed the strongest fluorescence intensities, and their relative fluorescence intensities (I/I_0_) were boosted 80- and 193-fold, respectively. Figure 2d shows that when *f*_w_ was 0 and 90/99, the compounds exhibited blue to bright yellow emissions. The absolute fluorescence quantum yields Φ_l_ (%) of these compounds were 1.51 and 49.26, respectively, in the maximum aggregation state in the system (Figure S7).

As presented in Figure 2e, the intensity of the aggregation emission peaks of the two compounds in H_2_O exceeded 200 times those in DMSO (I_577_/I_400_ and I_601_/I_440_). This finding indicated that TPAB-CF_3_ and TPAB-diCF_3_ had excellent AIE effects with increased poor solvents in the system and that they were typical AIE molecules. Figure 2f confirmed the excellent yellow fluorescence imaging ability of the compounds with increased poor solvents. TPAB-CF_3_ and TPAB-diCF_3_, as typical AIE molecules, had excellent bright yellow fluorescence emissions and had good potential application in the biological imaging field.

To further investigated the AIE feature detail of these compounds, the absorption and emission spectra of these compounds affected by the effect of viscosity was investigated in a glycerol/methanol system. As shown in Figure S8, with increasing glycerol fraction (*f*_g_/%), the maximum absorption peak of these compounds showed no obvious differences. With the *f*_g_ increasing from 0–80, the emission spectra of TPAB-CF_3_ gradually enhanced and reached the highest emission peak at 406 nm. For TPAB-diCF_3_, the emission spectra showed a likely phenomenon at 408 nm with *f*_g_ from 0–90. Moreover, when *f*_g_ was equal to 90 or 99, new aggregation peaks of the compound appeared at 569 and 583 nm. The results further confirmed that these compounds were AIE-type molecules.

The influence of pH is also very important in practical applications. PL spectra of the compounds were investigated under different pH conditions to observe the fluorescence peak positions and intensities. As shown in Figure S9, the compounds did not show obvious regular changes under different pH conditions. Except at pH ≤ 2, they displayed strong fluorescence emissions in neutral and weakly acidic microenvironments. These results indicated the potential application of the D-A structure AIE-type compounds with bright yellow fluorescence emissions in lysosome-targeted cell imaging experiments.

### 2.3. DFT Analysis of Compounds TPAB-CF_3_ and TPAB-diCF_3_

**Figure 3:**
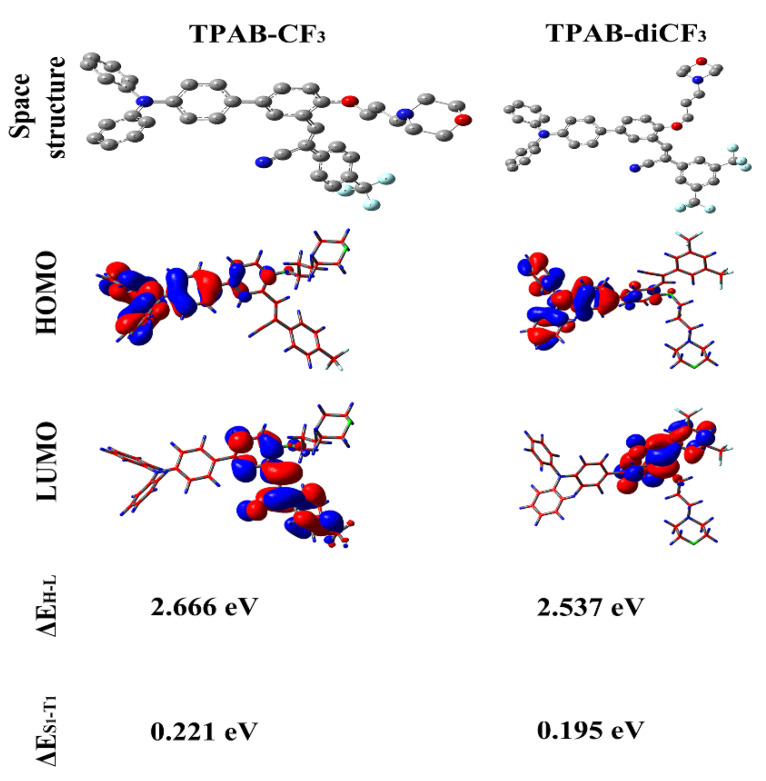
Molecular orbital amplitude plots of AIEgens based on DFT assay.

Given the excellent optical properties of these compounds, to further investigate the ICT effects, the electronic-cloud distribution characteristics of these compounds were analyzed by the DFT method. As shown in Figure 3, the highest occupied molecular orbital (HOMO) of the electron cloud was primarily distributed in the electron-donating groups triphenylamine (D_1_) and benzene ring (D_2_). Conversely, the lowest unoccupied molecular orbital (LUMO) was primarily located in the electron-withdrawing groups (4-(trifluoromethyl)benzeneacetonitrile or 3,5-bis(trifluoromethyl)benzeneacetonitrile). The ΔE_H−L_ of TPAB-CF_3_ and TPAB-diCF_3_ were 2.666 (HOMO:−4.973, LUMO: −2.307) and 2.537 eV (HOMO: −5.029, LUMO: −2.492), respectively, indicating that the ICT effect gradually strengthened and the electron cloud separation was good, consistent with the UV–vis spectral data. Meanwhile, the molecules with a strong D-A structure were more likely to achieve intersystem crossing (ISC) and lead to reduced difference in the singlet–triplet energy gap (ΔE_S1−T1_), in which the ΔE_S1−T1_ of TPAB-CF_3_ and TPAB-diCF_3_ were 0.221 (S_1_: 2.1473, T_1_: 1.9267) and 0.195 eV (S_1_: 2.0482, T_1_: 1.532), respectively (Table S1). This helped efficiently generate ROS, especially ^1^O_2_, under light irradiation [38]. It can also provide a theoretical basis for their application in PDT research.

### 2.4. Lysosome Imaging on HeLa Cells

**Figure 4:**
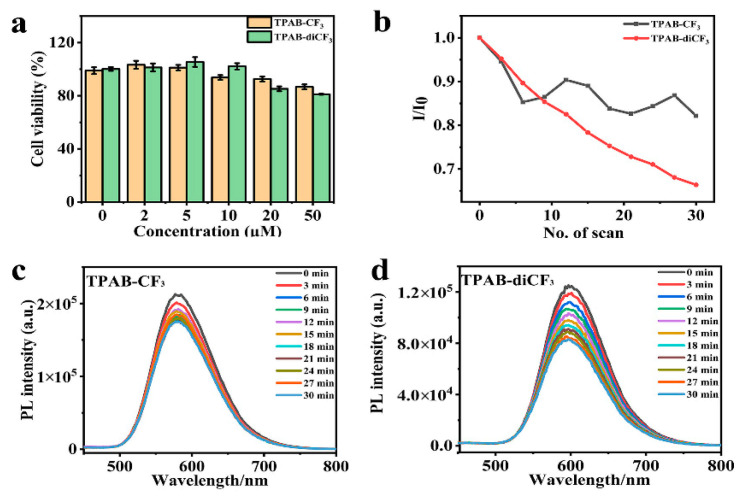
(**a**) Cell viability assessed by MTT assay of HeLa cells incubated with different concentrations of TPAB-CF_3_ and TPAB-diCF_3_ for 24 h. (**b**) Photobleaching of TPAB-CF_3_ and TPAB-diCF_3_ by increasing the number of sequential scans under light irradiation in the PBS solution. The PL spectra of (**c**) TPAB-CF_3_ and (**d**) TPAB-diCF_3_ (10 μM) in PBS solution with the same time interval.

In order to apply these compounds in cell imaging, the MTT method was implemented to estimate the biocompatibility of these compounds on HeLa cells. Figure 4a shows that when TPAB-CF_3_ reached 20 μM, the cell survival rate still reached 92%. However, at the same concentration of TPAB-diCF_3,_ the cell-survival rate was 85%, indicating it had certain toxicity to HeLa cells. These results showed that the working concentration (1 μM) of these AIEgens for staining and incubation with cells had good biocompatibility for the cells.

The photostability of these fluorescent dyes had great significance for the stable tracing of compounds in imaging samples. Figure 4c,d shows the samples after continuous irradiation with white light (30 mW/cm^2^) for 30 min in the PBS solution. Figure 4b shows that the fluorescence intensity of TPAB-CF_3_ decreased to 82% of the initial intensity, whereas that of TPAB-diCF_3_ decreased to 66%. Despite the existence of a certain degree of photobleaching, these AIEgens still had strong fluorescence emissions and could clearly mark the imaging targets.

**Figure 5:**
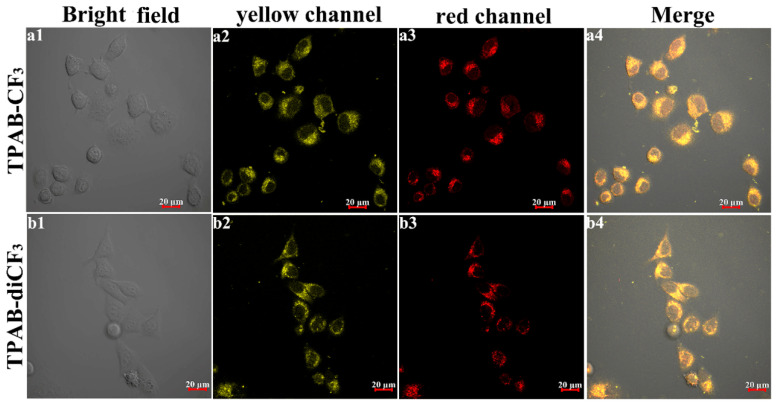
Co-localization images of HeLa cells incubated with DND-99 and AIEgens. (**a1**–**a4**) TPAB-CF_3_; (**b1**–**b4**) TPAB-diCF_3_. AIEgens: λ_ex_ = 405 nm, λ_em_ = 550–600 nm, and DND-99: λ_ex_ = 561 nm, λ_em_ = 570–630 nm. Dyes concentration: AIEgens (1 µM), DND-99 (100 nM). Scale bar: 20 µm.

The synthesized compounds had excellent optical properties and contained a weakly basic morpholine group, which provided a rich imagination for their targeting of acidic organelles through the “acid-base compatibility” principle, specifically lysosomes. Accordingly, we used the commercial lysosomal targeting dye LysoTracker Red DND-99 to evaluate the targeting ability of these AIEgens to lysosomes in subcellular organelles of HeLa cells via CLSM (Confocal Laser Scanning Microscope). After incubating HeLa cells with DND-99 (100 nM) and TPAB-CF_3_ or TPAB-diCF_3_ (1 µM) for 30 min, the cells were washed three times with PBS for confocal experiments. As presented in Figure 5, no background emission was displayed in the bright-field view (a1-b1). In the yellow and red channels, the probes and DND-99-stained HeLa cells were clearly observed to show bright yellow and red colors, respectively. The merged images (a4-b4) overlapped well, and the Pearson overlap correlation coefficients of TPAB-CF_3_ and TPAB-diCF_3_ with DND-99 were both 0.90 (Figure S10). These findings indicated that the AIEgens had an excellent lysosomal targeting ability with bright yellow emissions. They may have passed through the cell membrane by lipophilic interaction with the help of their appropriate LogP (the logarithmic value of the lipid–water partition coefficient) (TPAB-CF_3_: 9.49, TPAB-diCF_3_: 10.41) [28]. Second, due to the targeting ability of the weakly basic morpholine group in an acidic environment, the compounds more easily entered the acidic subcellular organelles and lysosomes through endocytosis. Hence, these AIEgens with bright yellow fluorescence emissions are excellent candidates for lysosome-specific targeting and labeling.

### 2.5. Photodynamic Therapy

These compounds have good biocompatibility and a remarkable ability to target lysosomes on HeLa cells. Meanwhile, AIEgens with a smaller ΔE_S1−T1_ are beneficial for promoting the generation of ROS under light irradiation. Thus, they may become an effective PS for PDT application by targeting lysosomes of cancer cells.

**Figure 6:**
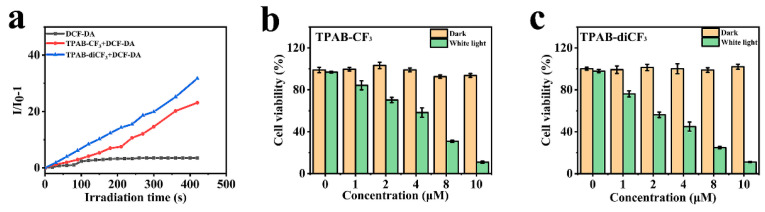
ROS generation upon white light irradiation and PDT study of all AIEgens to HeLa cells. (**a**) Relative PL intensity at 525 nm of DCF-DA in PBS solution. (**b**) Survival rate of HeLa co-incubated with TPAB-CF_3_. (**c**) TPAB-diCF_3_ under dark and light irradiation for 20 min.

In this experiment, the ROS-production ability of TPAB-CF_3_ and TPAB-diCF_3_ under light irradiation was evaluated by the enhancement in fluorescence peak at 525 nm when DCF-DA (2′,7′-Dichlorofluorescein) was oxidized [39]. As shown in Figure 6a, the relative fluorescence intensity of DCF-DA in PBS was slightly enhanced. As a contrast, the fluorescence intensities of the mixture of DCF-DA and TPAB-CF_3_ (TPAB-diCF_3_) increased by 23- and 31-fold, respectively, within 420 s under the same light irradiation times. This finding indicated that these AIEgens can effectively generate ROS as a PS, prompting us to explore their PDT effect on cancer cells.

As shown in Figure 6b,c, the cytotoxicity of TPAB-CF_3_ and TPAB-diCF_3_ on the model cancer cell HeLa was quantitatively evaluated using the MTT method. In a dark environment, TPAB-diCF_3_ showed almost no cytotoxicity toward HeLa cells at the tested concentrations, whereas TPAB-CF_3_ at the maximum concentration (10 µM) showed 7% cytotoxicity. Under light irradiation, the concentrations of TPAB-CF_3_ and TPAB-diCF_3_ reached 10 µM, and the survival rates of cancer cells were 11.1% and 10.8%, respectively. These results showed that the ROS production ability of these compounds under light irradiation was TPAB-diCF_3_ > TPAB-CF_3_, consistent with the theoretically calculated values of ΔE_S1−T1_.

**Figure 7:**
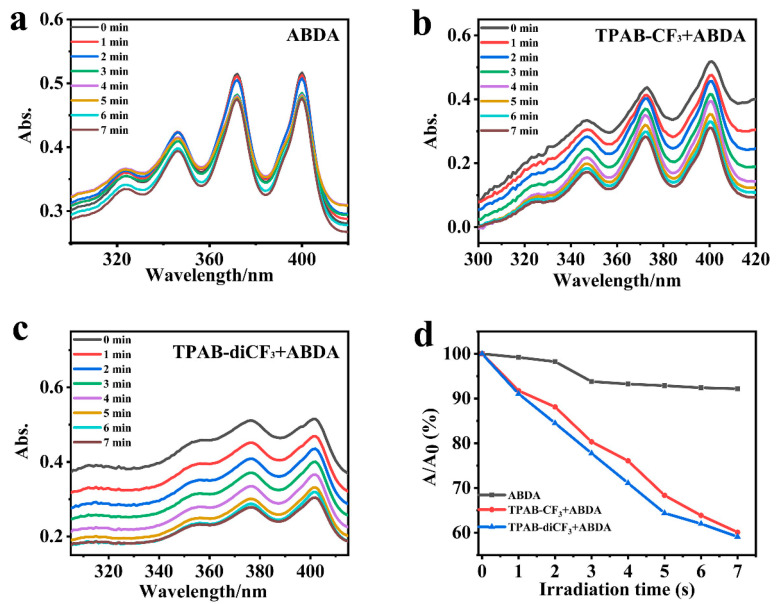
(**a**) Absorption spectra of ABDA absence or in the presence of (**b**) TPAB-CF_3_ or (**c**) TPAB-diCF_3_ under irradiation. (**d**) The relative decomposition rates of ABDA at 400 nm under light irradiation. Concentration: 10 µM (AIEgens), 50 µM (ABDA).

Among various ROS, ^1^O_2_ is deemed the main cytotoxic agent inducing cell apoptosis during PDT [40]. When ΔE_S1−T1_ was smaller, the generation of ^1^O_2_ was more favorable under irradiation. Accordingly, we used 9,10-anthryl-bis(methylenedi-1,3-diphosphonic acid) (ABDA) to assess the efficiency of these compounds in generating ^1^O_2_ under light irradiation. After ABDA was decomposed by ^1^O_2_, the characteristic absorption intensity of ABDA at 400 nm significantly decreased. As shown in Figure 7a–c, the absorbance of ABDA in PBS slightly decreased at 400 nm under white light irradiation. By contrast, the absorbance of ABDA in the presence of TPAB-CF_3_ and TPAB-diCF_3_ significantly decreased with the same irradiation time. As illustrated in Figure 7d, the slope of the degradation curve revealed that the decomposition rates of ABDA in the presence of TPAB-CF_3_ and TPAB-diCF_3_ were 40% and 41%, respectively, whereas the decomposition rate of ABDA was only 7.8% under the same conditions. The ^1^O_2_ generation capabilities of TPAB-CF_3_ and TPAB-diCF_3_ were calculated to be 8.41% and 10.9% in a PBS solution, respectively (Figure S11). These experimental results showed that the lysosome-targeting AIEgens can produce ROS under light irradiation, especially generating ^1^O_2_. At low concentrations, they can effectively perform the photodynamic killing of cancer cells, providing a rich imagination space for them to become an effective PDT therapeutic PS.

## 3. Materials and Methods

### 3.1. Materials

All the main raw chemicals, including 4-(diphenylamino)phenylboronicacid, 5-bromosalicylaldehyde, 4-(3-chloropropyl)morpholine, and 3,5-bis(trifluoromethyl)phe-nylacetonitrile, were purchased from Aladdin Chemical Reagent Company (Shanghai, China). All raw solvents were analytically pure.

### 3.2. Synthesis of the Probe TPAB-CF_3_ and TPAB-diCF_3_

#### 3.2.1. Synthesis of Precursor 1 and Precursor 2

Precursor 1 was synthesized according to the literature [41]. ^1^H NMR (600 MHz, CDCl_3_, TMS) δ(ppm): 11.13 (s, 1H), 9.96 (s, 1H), 7.79 (d, *J* = 8.52 Hz, 1H), 7.76 (d, *J* = 1.91 Hz, 1H), 7.52 (d, *J* = 8.34 Hz, 2H), 7.36 (t, *J* = 7.49 Hz, 4H), 7.24 (t, *J* = 7.75 Hz, 6H), 7.11–7.15 (m, 3H).

Precursor 2 was synthesized by the following steps. Specifically, precursor 1 (366.2 mg, 1.0 mmol) and anhydrous potassium carbonate (416.3 mg, 3.0 mmol) were dissolved in anhydrous acetonitrile (30 mL) in a round bottom flask. Under magnetic stirring, the reaction was refluxed for 2 h at 80 °C. After that, 4-(3-chloropropyl)morpholine (240 μL, 1.5 mmol) was added. The reaction continued for 4 h under the same conditions. Afterwards, the reaction was cooled to room temperature. The anhydrous acetonitrile was removed by vacuum distillation. The residue was extracted three times with dichloromethane and deionized water to remove excess potassium carbonate and residual 4-(3-chloropropyl)morpholine. After 4-(3-chloropropyl)morpholine removal, dichloromethane was removed, and the yellow oily precursor 2 was obtained. ^1^H NMR (600 MHz, CDCl_3_, TMS) δ(ppm): 10.56 (s, 1H), 8.07 (d, *J* = 2.05 Hz, 1H), 7.77 (d, 8.53 Hz, 1H), 7.47 (d, *J* = 8.41 Hz, 2H), 7.30 (s, 1H), 7.28 (d, *J* = 3.32 Hz, 2H), 7.15 (d, *J* = 8.21 Hz, 7H), 7.09 (d, *J* = 8.74 Hz, 1H), 7.06 (t, *J* = 6.93 Hz, 2H), 4.22 (t, *J* = 6.08 Hz, 2H), 3.77 (s, 4H), 2.61 (t, *J* = 7.08 Hz, 2H), 2.52 (s, 4H), 2.10 (t, *J* = 6.61 Hz, 2H).

#### 3.2.2. Synthesis of TPAB-CF_3_

Precursor 2 (1.0 mmol) and 2-(4-(trifluoromethyl)phenyl)acetonitrile (0.222 mg, 1.2 mmol) were mixed in ethanol (50 mL), and the reaction was reacted for 3 h under magnetic stirring at 80 °C, Afterwards, the reaction was cooled to room temperature. The anhydrous ethanol was removed by vacuum distillation. The residue was dissolved in dichloromethane. The mixture was purified via silica column chromatography (CH_2_Cl_2_:MeOH = 40:1), and a snowflake-like yellow powder (TPAB-CF_3_) was obtained (61%). ^1^H NMR (600 MHz, CDCl_3_, TMS) δ(ppm): 8.41 (s,1H), 8.10 (s,1H), 7.84 (d, *J* = 8.05 Hz, 2H), 7.75 (d, *J* = 8.12 Hz, 2H), 7.66 (d, *J* = 8.56 Hz, 1H), 7.53 (d, *J* = 8.40 Hz, 2H), 7.31 (d, *J* = 7.87 Hz, 4H),7.17 (t, *J* = 7.31 Hz, 6H), 7.06 (t, *J* = 7.88 Hz, 3H), 4.19 (t, *J* = 6.38 Hz, 2H), 3.75 (s, 4H), 2.58 (t, *J* = 6.47Hz, 2H), 2.50 (s, 4H), 2.09 (t, *J* = 6.43 Hz, 2H). HRMS(ESI) [M + H]^+^ for C_41_H_36_F_3_N_3_O_2_ calcd: 660.28324, found: 660.28182.

#### 3.2.3. Synthesis of TPAB-diCF_3_

The synthesis steps of TPAB-diCF_3_ were similar to those of TPAB-CF_3_, and a deep yellow solid was obtained (63%). ^1^H NMR (600 MHz, CDCl_3_, TMS) δ(ppm): 8.40 (s, 1H), 8.12 (s, 1H), 7.92 (s, 1H), 7.70 (d, *J* = 8.57 Hz, 1H), 7.52 (d, *J* = 8.41 Hz, 2H), 7.30 (t, *J* = 7.54 Hz, 6H), 7.17 (t, *J* = 7.88 Hz, 5H), 7.06 (t, *J* = 7.55 Hz, 3H), 4.22 (t, *J* = 6.22 Hz, 2H), 3.76 (s, 4H), 2.59 (s, 3H), 2.50 (s, 3H), 2.10 (s, 2H). HRMS(ESI) for C_41_H_36_F_3_N_3_O_2_ calcd: 728.27062, found: 728.27098.

### 3.3. Spectral Measurement

The stock solutions of TPAB-CF_3_ and TPAB-diCF_3_ were prepared in DMSO (10 mM). All of the spectral measurements were carried out at a synthesized compound concentration of 10 µM.

### 3.4. DFT Calculations

Firstly, the optimized structures of TPAB-CF_3_ and TPAB-diCF_3_ were obtained by Chem3D Pro 14.0. Secondly, the optimized molecules were calculated based on the B3LYP/6-31G(d) method by using Gaussian 09W software and the DFT method. Finally, the corresponding E_HOMO_ and E_LUMO_ as well as the energy difference (ΔE_S1−T1_) were obtained.

### 3.5. Cytotoxicity and Cellular Imaging

The cytotoxicity of the two compounds toward Hela cells was investigated via MTT assay. The Hela cells were first inoculated in a culture plate and passed down until they stuck to the walls. Then, Hela cells were seeded into 96-well cell culture plates at a density of about 6000 cells per well for 12 h. Subsequently, different concentrations (0, 2, 5, 10, 20 and 50 µM) of AIEgens (i.e., TPAB-CF_3_ and TPAB-diCF_3_) were added into the RPMI-1640 medium and cultured in a humidified incubator with 5% CO_2_ at 37 °C for 24 h. The medium was removed, and MTT (10 µL) and a fresh medium (1990 µL) were added into each well. After storing in the dark for another 4 h, the supernatant was discarded, and a certain amount of DMSO was added to dissolve the precipitation. Finally, the plate was lightly shaken, and the absorbance was recorded on a microplate reader.

For this test, Hela cells with well-developed morphology were seeded into 20 mm glass cell culture dishes. Then, they were incubated in the presence of the compounds TPAB-CF_3_ and TPAB-diCF_3_ (1 µM, final concentration of DMSO < 0.1%) and LysoTracker Red DND-99 (100 nM) in a PBS buffer solution for 30 min. The dishes were washed and placed under a laser confocal microscope for imaging.

### 3.6. ROS and ^1^O_2_ Production and Phototoxicity of AIEgens on Hela Cells Under Irradiation

H2DCF-DA and ABDA were chosen as indicators to detect ROS and ^1^O_2_. In the ROS generation experiment, raw H2DCF-DA powder was dissolved in the PBS solution. After a sodium hydroxide solution was added, DCF-DA was prepared at a concentration of 40 μM. The synthesized AIEgens solution (20 μL, 1 mM) was then added homogeneously to the DCF-DA PBS solution. Once the DCF-DA solution was oxidized, the fluorescence peak at 525 nm was recorded. In the ^1^O_2_ generation experiment, a certain amount of ABDA was weighed and diluted to prepare a 7.5 mM PBS stock solution, which was placed in a dark room for future use. The TPAB-CF_3_ or TPAB-diCF_3_ solution (20 μL, 10 mM) and ABDA stock solution (13.3 μL, 7.5 mM) were added homogeneously to the 2 mL PBS buffer solution. They were mixed evenly and irradiated under a xenon lamp (light intensity: 30 mW/cm^2^). The absorption intensity at 400 nm was measured and recorded using a UV–visible spectrometer.

Hela cells were cultured in a 96-well plate at a density of approximately 6000 cells. After 24 h, a fresh medium (100 μL) containing different concentrations of synthesized solution was added to replace the old medium. After being cultured for 20 min, the plate was irradiated for 20 min under the xenon lamp at a intensity of 30 mW/cm^2^, and another plate was placed in the dark with no treatment to serve as the control group.

## 4. Conclusions

Two novel triphenylamine-based compounds with bright yellow fluorescence emissions, namely, TPAB-CF_3_ and TPAB-diCF_3_, were designed and synthesized. Compared to some reported AIEgens with dual functions of lysosome imaging and PDT application [30,31,42,43,44,45,46] (Table S2), these compounds exhibited 80- and 193-fold fluorescence intensity increases, which were the most obvious AIE phenomenon in the DMSO/H_2_O system. Especially for TPAB-diCF_3_, the quantum yield was 49.26% in the PBS solution. The larger stocks shifts of compounds were 177 nm and 160 nm, which are conducive to their observation in an aqueous solution environment. Moreover, these two AIEgens with a D_1_-D_2_-π-A structure were formed by virtue of the twisted spatial structure and strong electron-donating characteristics of triphenylamine, through the bridging of phenyl groups, and by the combination of different electron-withdrawing groups. These molecules had good biocompatibility and proper optical stability. Meanwhile, with the aid of certain lipophilicity and a weakly basic morpholine ring, they can enter and light up lysosomes in cancer cells with bright yellow emissions. Furthermore, the narrow bandgap of the molecules enabled the effective separation of the electron cloud in the HOMO-LUMO of the molecules. It enhanced the ISC process and greatly reduced the ΔE_S1−T1_, which effectively promoted the generation of ^1^O_2_ under white light irradiation. The outcome was the effective photodynamic killing of model cancer cells. This work enriches the research on lysosome-targeting AIEgens and provides potential PS selection for PDT research.

## Data Availability

Data are contained within the article and Supplementary Materials.

## Supplementary Materials

The following supporting information can be downloaded at: https://www.mdpi.com/article/10.3390/molecules30112272/s1, Figure S1: The ^1^H NMR of precusor 1; Figure S2: The ^1^H NMR of precusor 2; Figure S3: The ^1^H NMR of TPAB-CF_3_; Figure S4: The HRMS of TPAB-CF_3_; Figure S5: The ^1^H NMR of TPAB-diCF_3_; Figure S6: The HRMS of TPAB-diCF_3_; Figure S7: The quantum yields of TPAB-CF_3_ and TPAB-diCF_3_ in DMSO/H_2_O; Figure S8: The normalized absorption and emission spectra of TPAB-CF_3_ and TPAB-diCF_3_ in methanol/glycerol system; Figure S9: The PL spectra of (A) TPAB-CF_3_ (B) TPAB-diCF_3_ (10 μM) in different pH PBS solution; Figure S10: Scatter plot of each AIEgen (A) TPAB-CF_3_, (B) TPAB-diCF_3_ and DND-99 in living Hela cells. Pearson correlation coefficients Rr = 0.90; Figure S11: Photodegradation of ABDA with RB (A), TPAB-CF_3_ (D), and TPAB-diCF_3_ (G). The absorption peak area of RB (B), TPAB-CF_3_ (E), and TPAB-diCF_3_ (H); The decomposition rate constants of RB (C), TPAB-CF_3_ (F), and TPAB-diCF_3_ (I); Table S1: The calculations and data of the DFT analysis of the compounds; Table S2: Photophysical properties of lysosomes probes and their applications. [30,31,42,43,44,45,46,47].

## References

[1] Luzio J.P., Pryor P.R., Bright N.A. (2007). Lysosomes: Fusion and function. Nat. Rev. Mol. Cell Biol..

[2] Rebecca V.W., Nicastri M.C., McLaughlin N., Fennelly C., McAfee Q., Ronghe A., Nofal M., Lim C.-Y., Witze E., Chude C.I. (2017). A unified approach to targeting the lysosome’s degradative and growth signaling roles. Cancer Discov..

[3] Cesen M.H., Pegan K., Spes A., Turk B. (2012). Lysosomal pathways to cell death and their therapeutic applications. Exp. Cell Res..

[4] Lawrence R.E., Zoncu R. (2019). The lysosome as a cellular centre for signalling, metabolism and quality control. Nat. Cell Biol..

[5] He Y., Li W.M., Liao G.J., Xie J.P. (2012). Mycobacterium tuberculosis-specific phagosome proteome and underlying signaling pathways. J. Proteome Res..

[6] Oyarzun J.E., Lagos J., Vazquez M.C., Valls C., De la Fuente C., Yuseff M.I., Alvarez A.R., Zanlungo S. (2019). Lysosome motility and distribution: Relevance in health and disease. Biochim. Biophys. Acta Mol. Basis Dis..

[7] Devany J., Chakraborty K., Krishnan Y. (2018). Subcellular nanorheology reveals lysosomal viscosity as a reporter for Lysosomal storage diseases. Nano Lett..

[8] Boyd R.E., Lee G., Rybczynski P., Benjamin E.R., Khanna R., Wustman B.A., Valenzano K.J. (2013). Pharmacological chaperones as therapeutics for lysosomal storage diseases. J. Med. Chem..

[9] Appelqvist H., Wäster P., Kågedal K., Öllinger K. (2013). The lysosome: From waste bag to potential therapeutic target. J Mol. Cell Biol..

[10] Kallunki T., Olsen O.D., Jäättelä M. (2013). Cancer-associated lysosomal changes: Friends or foes?. Oncogene.

[11] Gyparaki M.T., Papavassiliou A.G. (2014). Lysosome: The cell’s ‘suicidal bag’ as a promising cancer target. Trends Mol. Med..

[12] Tsubone T.M., Martins W.K., Pavani C., Junqueira H.C., Itri R., Baptista M.S. (2017). Enhanced efficiency of cell death by lysosome-specific photodamage. Sci. Rep..

[13] Zhou K., Yu Y., Xu L.T., Wang S.Y., Li Z.J., Liu Y., Kwok R.T.K., Sun J., Lam J.W.Y., He G. (2024). Aggregation-induced emission luminogen based wearable visible-light penetrator for deep photodynamic therapy. ACS Nano.

[14] Cheng P.H., Pu K.Y. (2020). Activatable Phototheranostic materials for imaging-guided cancer therapy. ACS Appl. Mater. Interfaces.

[15] Zuo Y.F., Shen H.C., Sun F.Y., Li P., Sun J.W., Kwok R.T.K., Lam J.W.Y., Tang B.Z. (2022). Aggregation-induced emission luminogens for cell death research. ACS Bio Med. Chem. Au.

[16] Murali G., Kwon B., Kang H., Modigunta J.K.R., Park S., Lee S., Lee H., Park Y.H., Kim Y.J., Park S.Y. (2022). Hematoporphyrin photosensitizer-linked carbon quantum dots for photodynamic therapy of cancer cells. ACS Appl. Nano Mater..

[17] Usama S.M., Thavornpradit S., Burgess K. (2018). Optimized heptamethine cyanines for photodynamic therapy. ACS Appl. Bio Mater..

[18] Liu M., Wang C.J., Qian Y. (2021). Novel indole-BODIPY photosensitizers based on iodine promoted intersystem crossing enhancement for lysosome-targeted imaging and photodynamic therapy. New J. Chem..

[19] Yang N., Song S., Ren J., Liu C., Li Z.H., Qi H., Yu C. (2021). Controlled aggregation of a perylene-derived probe for near Infrared fluorescence imaging and phototherapy. ACS Appl. Bio Mater..

[20] Bandyopadhyay S., Forzano J.A., Dirak M., Chan J. (2025). Activatable porphyrin-based sensors, photosensitizers and combination therapeutics. JACS Au.

[21] Ma X.F., Sun R., Cheng J.H., Liu J.Y., Gou F., Xiang H.F., Zhou X.G. (2016). Fluorescence aggregation-caused quenching versus aggregation induced emission: A visual teaching technology for undergraduate chemistry students. J. Chem. Educ..

[22] Wu W.B., Shi L.L., Duan Y.K., Xu S.D., Gao X.H., Zhu X.Y., Liu B. (2021). Metabolizable photosensitizer with aggregation-induced emission for photodynamic therapy. Chem. Mater..

[23] Lou J., Xie Z., Lam J.W.Y., Cheng L., Chen H., Qiu C., Kwok H.S., Zhan X.W., Liu Y.Q., Zhu D.B. (2001). Aggregation-induced emission of 1-methyl-1,2,3,4,5-pentaphenylsilole. Chem. Commun..

[24] Wang W.J., Xin Z.Y., Su X.X., Hao L., Qiu Z.J., Li K., Luo Y.M., Cai X.-M., Zhang J.Q., Alam P. (2025). Aggregation-induced emission luminogens realizing high-contrast bioimaging. ACS Nano.

[25] Tang Y.X., Cao Y., Shi W.J., Li J.C., Lu W.L., Fan T., Zheng L., Yan J.-W., Han D., Niu L. (2024). Construction of cationic meso-thiazolium BODIPY AIE fluorescent probes for viscosity imaging in dual organelles. Chem. Commun..

[26] Ingle J., Basu S. (2023). Mitochondria targeted AIE probes for cancer phototherapy. ACS Omega.

[27] Sun Z.G., Shi S.M., Guan P.L., Liu B. (2022). Construction of heteroaryl-bridged NIR AIEgens for specific imaging of lipid droplets and its application in photodynamic therapy. Spectrochim. Acta Part A Mol. Biomol. Spectrosc..

[28] Zhang F., Zhang Y., Li Z.X., Wu X.X., Wang D., He Y.L., Cheng H., Fan B.L., Zhu D., Li M. (2024). Engineered strategies for lipid droplets-targeted AIEgens. Molecules.

[29] Peng Y., Mo R.F., Yang M.W., Xie H.L., Ma F.L., Ding Z.Y., Wu S., Lam J.W.Y., Du J., Zhang J.Q. (2024). Mitochondria-targeting AIEgens as pyroptosis inducers for boosting Type-I photodynamic therapy of tongue squamous cell carcinoma. ACS Nano.

[30] Dai Y.P., He F.R., Ji H.F., Zhao X.X., Misal S., Qi Z.J. (2020). Dual-functional NIR AIEgens for high-fidelity imaging of lysosomes in cells and photodynamic therapy. ACS Sens..

[31] Huang W.D., Zhang Y.J., Tan X.Y., Wang N., Wang J.H., He M.L., Peng J., Hu J., Zhao Y., Wang S. (2021). An AIEgen-based photosensitizer for lysosome imaging and photodynamic therapy in tumor. Sens. Actuators B Chem..

[32] Roncali J. (1997). Synthetic principles for bandgap control in linear π-conjugated systems. Chem. Rev..

[33] Zhao X., Liu J., Fan J., Chao H., Peng X. (2021). Recent progress in photosensitizers for overcoming the challenges of photodynamic therapy: From molecular design to application. Chem. Soc. Rev..

[34] Wei D., Chen Y., Huang Y., Li P., Zhao Y., Zhang X., Wan J., Yin X.Y., Liu T., Yin J.Y. (2021). NIR light triggered dual-cascade targeting core-shell nanoparticles enhanced photodynamic therapy and immunotherapy. Nano Today.

[35] Wang R., Li X.S., Yoon J.Y. (2021). Organelle-targeted photosensitizers for precision photodynamic therapy. ACS Appl. Mater. Interfaces.

[36] Lu H.G., Zheng Y.D., Zhao X.W., Wang L.J., Ma S.Q., Han X.Q., Xu B., Tian W.J., Gao H. (2016). Highly efficient far red/near-infrared solid fluorophores: Aggregation-induced emission, intramolecular charge transfer, twisted molecular conformation, and bioimaging applications. Angew. Chem. Int. Ed..

[37] Gu X.G., Zhao E.G., Lam J.W.Y., Peng Q., Xie Y.J., Zhang Y.L., Wong K.S., Sung H.H., Williams I.D., Tang B.Z. (2015). Mitochondrion-specific live-cell bioprobe operated in a fluorescence turn-on manner and a well-designed photoactivatable mechanism. Adv. Mater..

[38] Li J., Zhuang Z.Y., Zhao Z.J., Tang B.Z. (2021). Type I AIE photosensitizers: Mechanism and application. View.

[39] Qi J.Q., Ou H., Liu Q., Ding D. (2021). Gathering brings strength: How organic aggregates boost disease phototheranostics. Aggregate.

[40] DeRose M.C., Crutchley R.J. (2002). Photosensitized singlet oxygen and its applications. Coord. Chem. Rev..

[41] Li Y.L., Ren T.H., Dong W.J. (2013). Tuning photophysical properties of triphenylamine and aromatic cyano conjugate-based wavelength-shifting compounds by manipulating intramolecular charge transfer strength. Photochem. Photobiol. A.

[42] Zhuang J.B., Yang H.X., Li Y., Wang B., Li N., Zhao N. (2020). Efficient photosensitizers with aggregation-induced emission characteristics for lysosome- and Gram-positive bacteria-targeted photodynamic therapy. Chem. Commun..

[43] Deng J.R., Yang M.Q., Li C., Liu G.Y., Sun Q., Luo X.G., Wu F.S. (2021). Single molecular-based nanoparticles with aggregation-induced emission characteristics for fluorescence imaging and efficient cancer phototherapy. Dyes Pigments.

[44] Zhou Y.P., Zhang D., He G.H., Liu C., Tu Y.N., Li X., Zhang Q.B., Wu X., Liu R.Y. (2021). A lysosomal targeted NIR photosensitizer for photodynamic therapy and two-photon fluorescence imaging. J. Mater. Chem. B.

[45] Liao Y.H., Wang R.L., Wang S.Z., Xie Y.F., Chen H.H., Huang R.J., Shao L.Q., Zhu Q.H., Liu Y.S. (2021). Highly efficient multifunctional organic photosensitizer with Aggregation-Induced Emission for In vivo bioimaging and photodynamic therapy. ACS Appl. Mater. Interfaces.

[46] Huang L., Qing D.Y., Zhao S.J., Wu X.L., Yang K., Ren X.J., Zheng X.L., Lan M.H., Ye J., Zeng L.T. (2022). Acceptor-donor-acceptor structured deep-red AIE photosensitizer: Lysosome-specific targeting, in vivo long-term imaging, and effective photodynamic therapy. Chem. Eng. J..

[47] Wu W., Mao D., Xu S., Ji S., Hu F., Ding D., Kong D., Liu B. (2017). High performance photosensitizers with aggregation-induced emission for image-guided photodynamic anticancer therapy. Mater. Horiz..

