# Supplementary material for: Construction of Triphenylamine-Based Aggregation-Induced Emission Luminogens for Lysosomes Imaging and Its Application in the Photodynamic Therapy of Cancer Cells
Source: Molecules. 2025 May 22;30(11):2272. doi: 10.3390/molecules30112272 (PMC12155880; doi:10.3390/molecules30112272)
Supplement: Supplementary file 1 [file molecules-30-02272-s001.zip › molecules-3607610-supplementary.pdf]

Supporting Information

Construction of Triphenylamine-Based Aggregation-Induced Emission Luminogens for Lysosomes Imaging and Its Application in the Photodynamic Therapy of Cancer Cells

Zhanguo Sun [1,*], Bin Liu [2] and Huijun Liu [1]

[1] School of Chemistry and Chemical Engineering, Shanxi Datong University, Datong 037009, China; dtdxlhj@163.com
[2] Key Laboratory of Chemical Biology and Molecular Engineering, Ministry of Education, Institute of Molecular Science, Shanxi University, Taiyuan 030006, China; liubin@sxu.edu.cn
* Correspondence: sunzhanguo1314@163.com

Table of contents

Figure. S1–S6. ^{1}H NMR and MS of compounds.

Figure S7. The quantum yields of TPAB-CF_3 and TPAB-diCF_3 in the maximum aggregation state in DMSO/H_2O system.

Figure S8. The normalized absorption and emission spectra of TPAB-CF_3 and TPAB-diCF_3 in methanol/glycerol system.

Figure S9. The PL spectra of (A) TPAB-CF_3 (B) TPAB-diCF_3 (10 μM) in different pH PBS solution.

Figure S10. Scatter plot of AIEgens and DND-99 in Hela cell -colocalization imaging.

Figure S11. Photodegradation of ABDA with RB (A), TPAB-CF_3 (D), and TPAB-diCF_3 (G). The absorption peak area of RB (B), TPAB-CF_3 (E), and TPAB-diCF_3 (H); The decomposition rate constants of RB (C), TPAB-CF_3 (F), and TPAB-diCF_3 (I).

$^1O_2$ quantum yield measurements.

Table S1. The calculations and data of the DFT analysis of the compounds

Table S2. The features and applications of those AIEgens compared with the reported works.

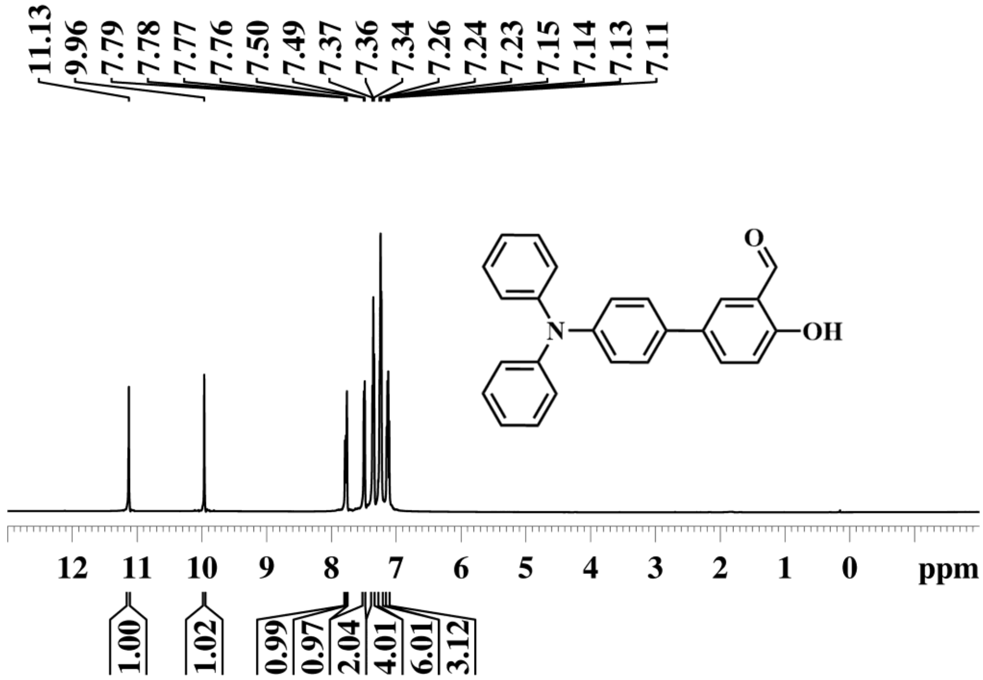

Figure S1. The ^{1}H NMR of precusor 1. Solvent: $CDCl_3$.

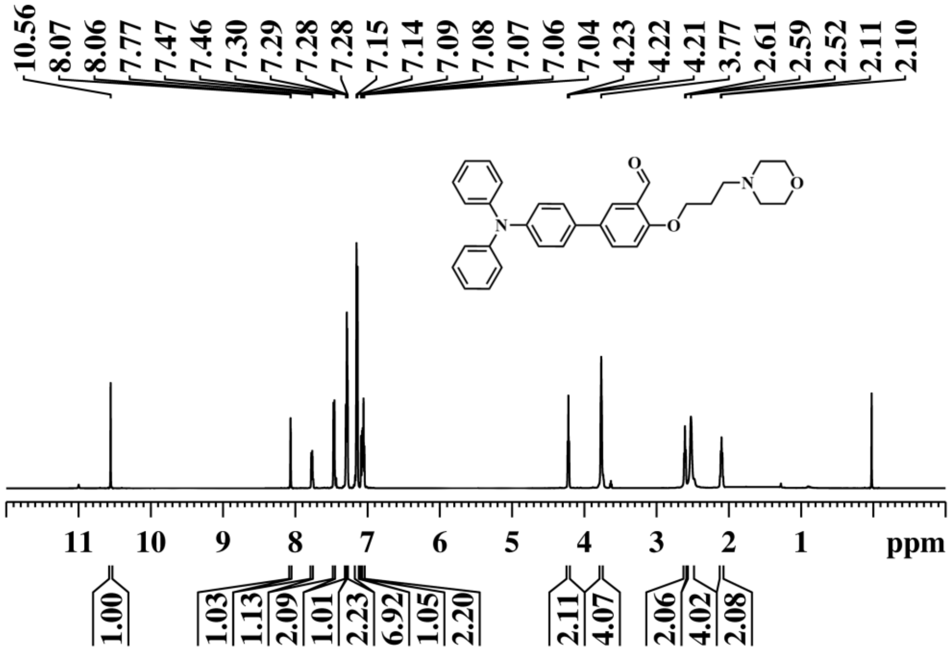

Figure S2. The ^{1}H NMR of precusor 2. Solvent: $CDCl_3$.

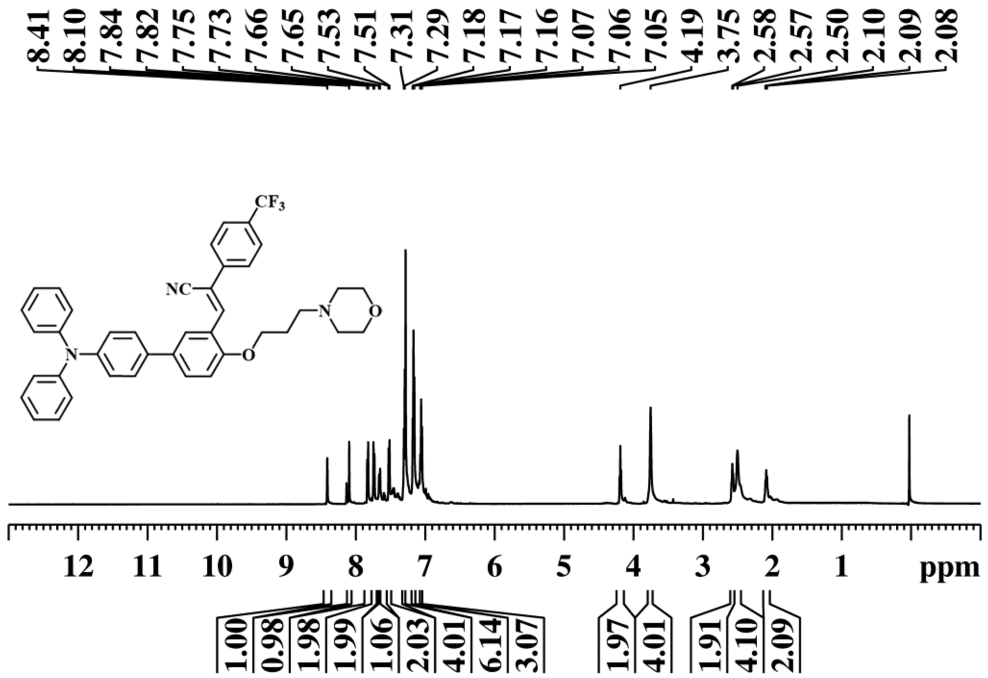

Figure S3. The ^{1}H NMR of TPAB-CF_3. Solvent: $CDCl_3$.

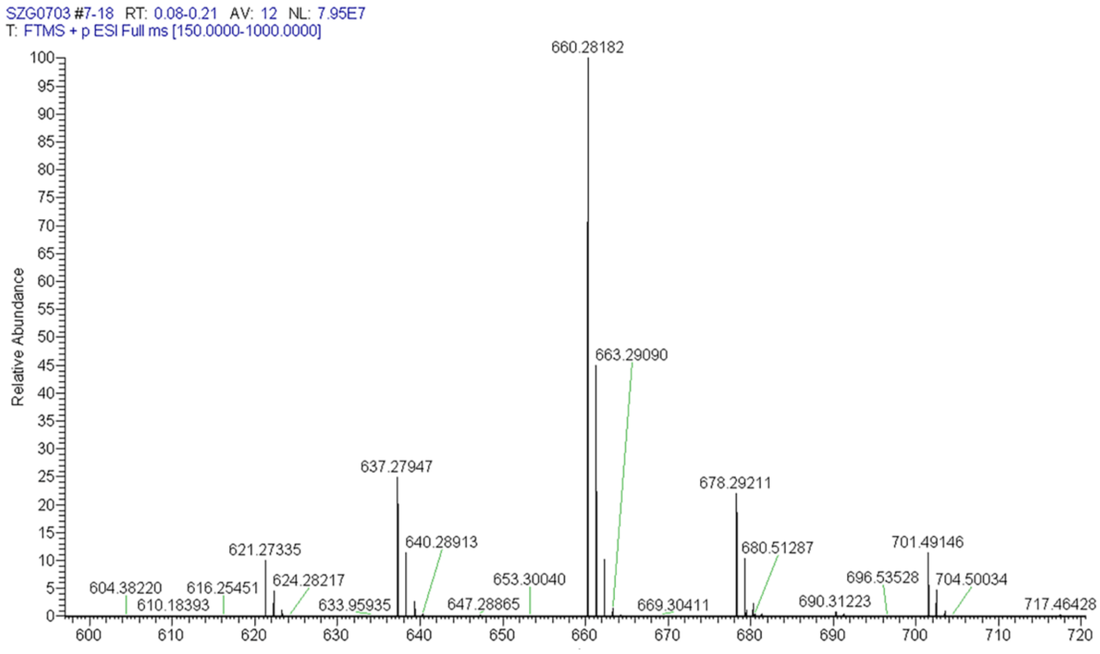

Figure S4. The HRMS of TPAB-CF_3.

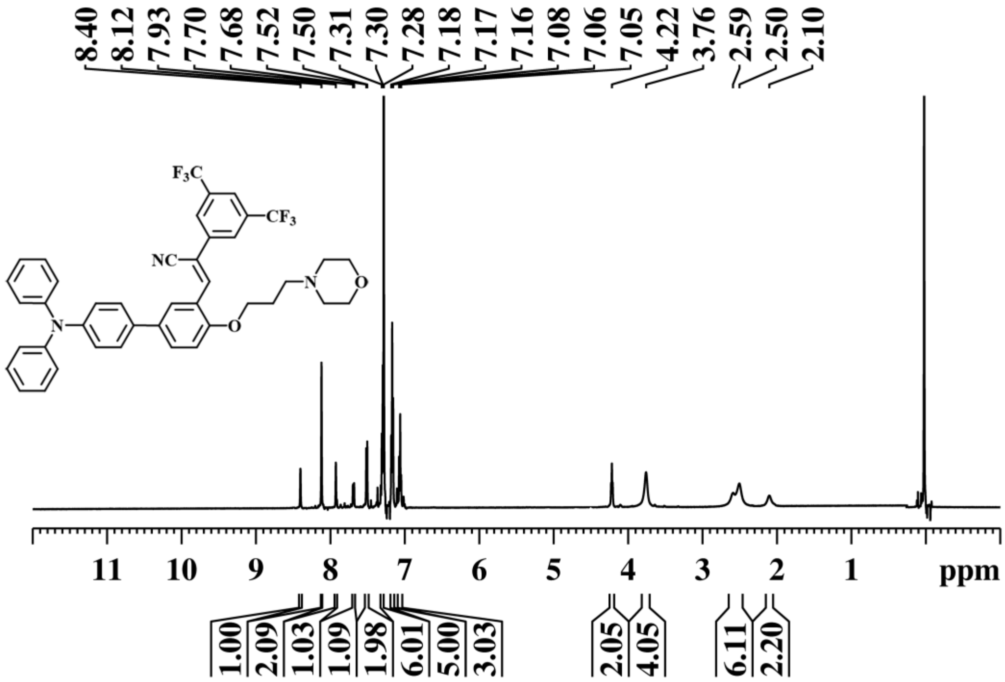

Figure S5. The ¹H NMR of TPAB-diCF3. Solvent: CDCl3.

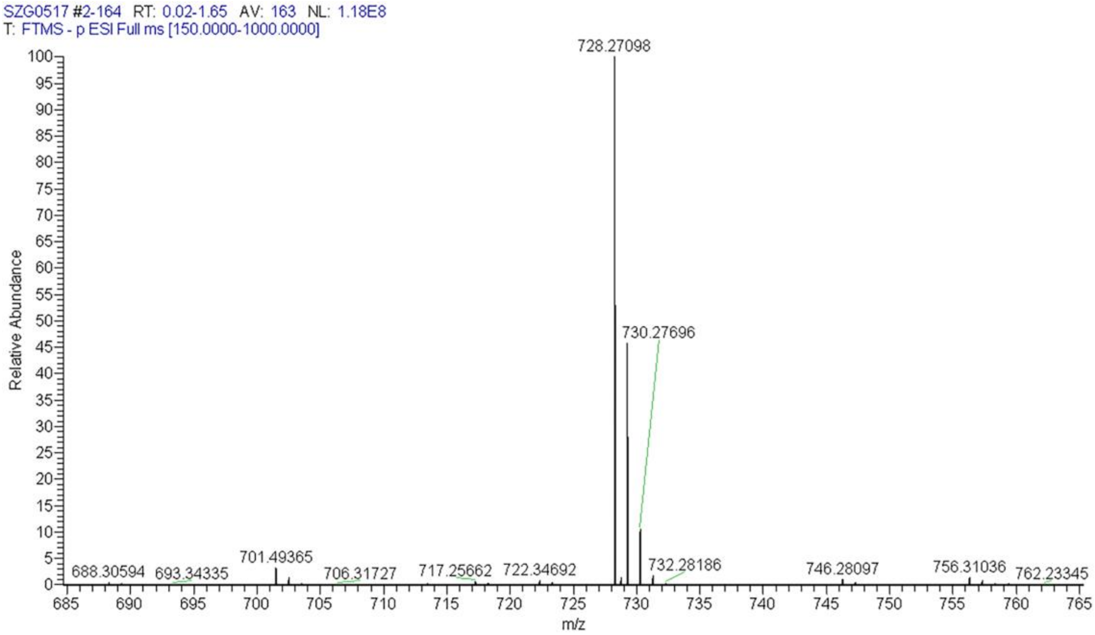

Figure S6. The HRMS of TPAB-diCF3.

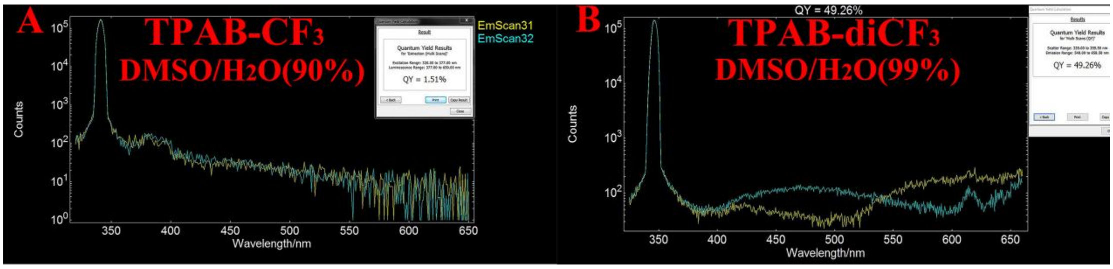

Figure S7. The quantum yields of TPAB-CF_3 and TPAB-diCF_3 in DMSO/H_2O.

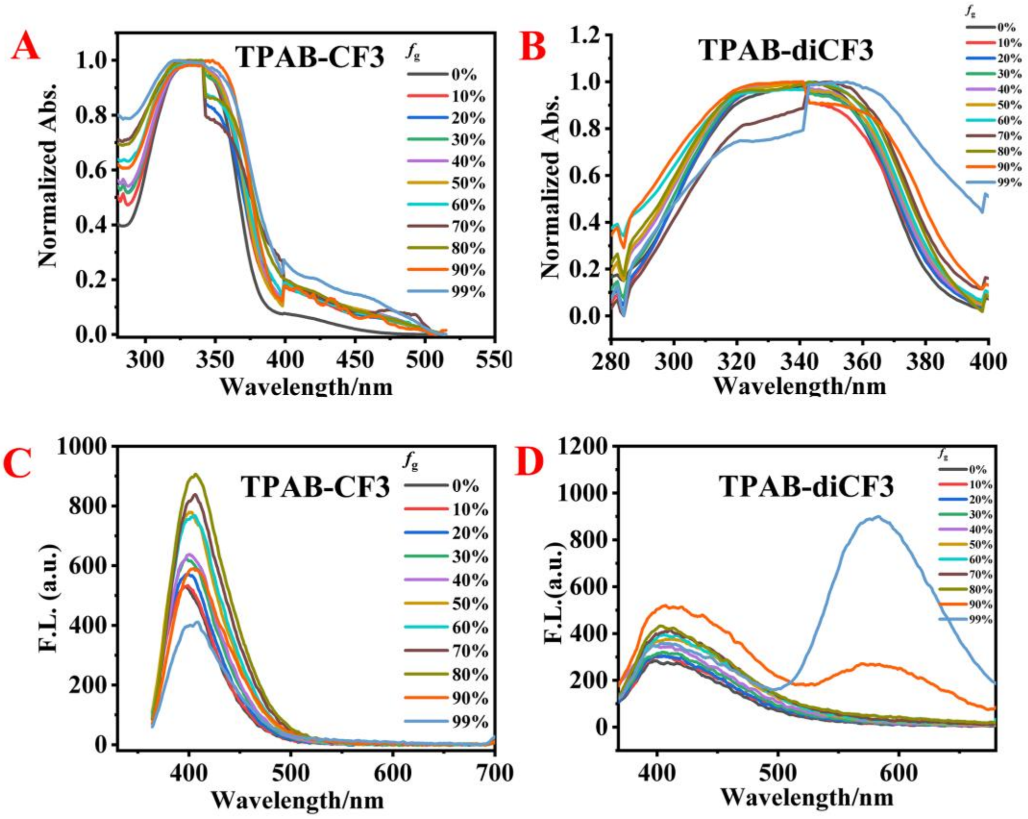

Figure S8. The normalized absorption and emission spectra of TPAB-CF_3 and TPAB-diCF_3 in methanol/glycerol system.

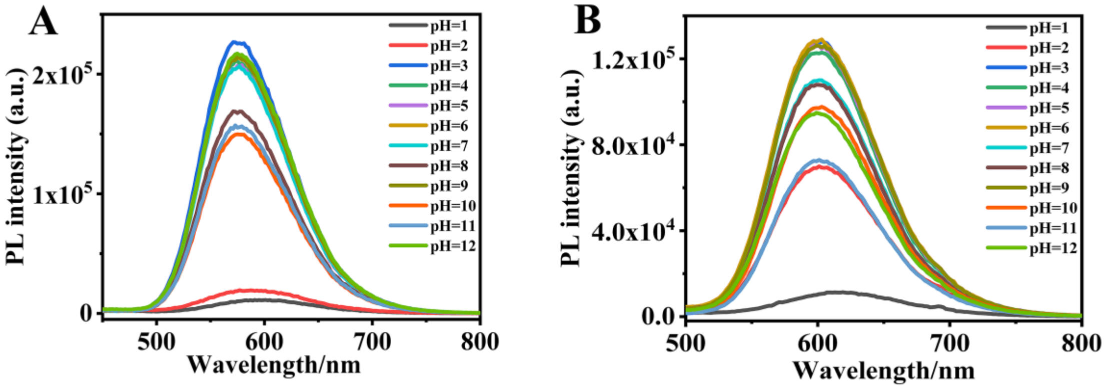

Figure S9. The PL spectra of (A) TPAB-CF_3 (B) TPAB-diCF_3 (10 μM) in different pH PBS solution.

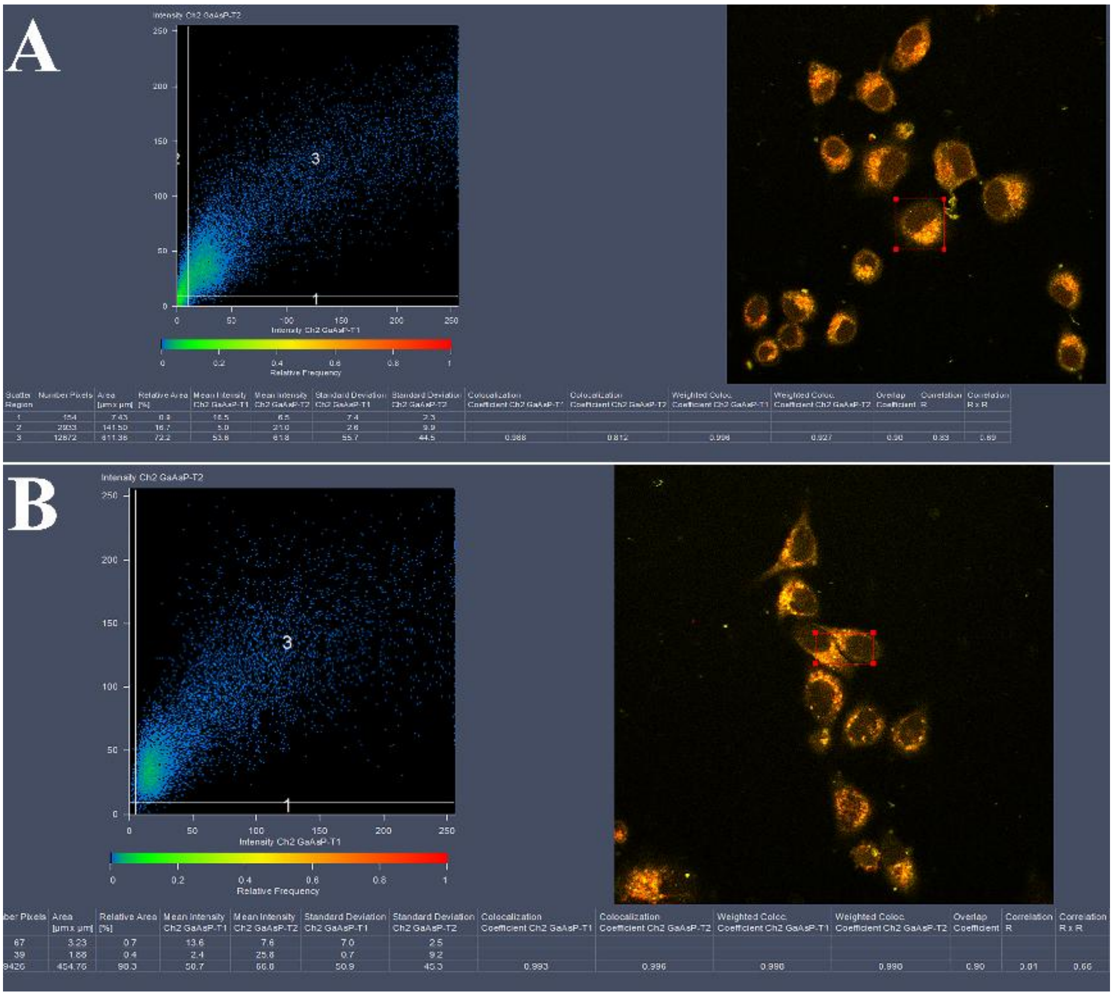

Figure S10. Scatter plot of each AIEgen (A) TPAB-CF_3, (B) TPAB-diCF_3 and DND-99 in living Hela cells. Pearson correlation coefficients Rr = 0.90.

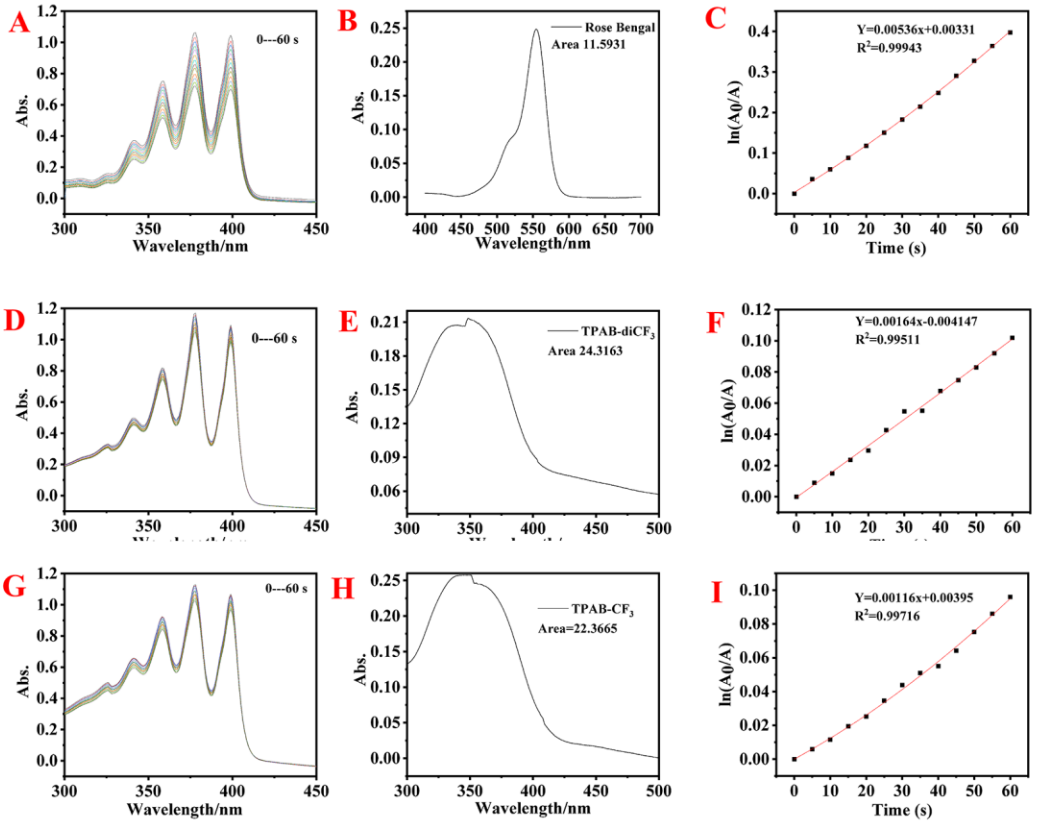

Figure S11. Photodegradation of ABDA with RB (A), TPAB-CF_3 (D), and TPAB-diCF_3 (G). The absorption peak area of RB (B), TPAB-CF_3 (E), and TPAB-diCF_3 (H); The decomposition rate constants of RB (C), TPAB-CF_3 (F), and TPAB-diCF_3 (I).

$^1O_2$ quantum yield measurements[1].

The ABDA was used as $^1O_2$-sensitive indicator, and Rose Bengal (RB) was employed as the standard photosensitizer. In general, the ABDA solution was added into sample solution (100 μM), and the white light (30 mW cm^{-2}) was used as the irradiation source. The absorbance of ABDA at 400 nm was recorded at different irradiation times to obtain the decay rate of the photo-sensitizing process. The $^1O_2$ quantum yield of the desired PS in water was calculated using the following formula :

$$\Phi_{ps}=\Phi_{RB}\ K_{PS}A_{RB}/K_{RB}A_{PS}$$

where K_{PS} and K_{RB} represent the decomposition rate constants of ABDA by the TPAB-CF_3 (or TPAB-diCF_3) and RB, respectively. A_{PS} and A_{RB} represent the light absorbed by the TPAB-CF_3 (or

TPAB-diCF$_3$) and RB, respectively, and were determined by integrating of the areas under the absorption bands in the wavelength range of 400-600 nm (RB) or300-500 nm (TPAB-CF$_3$ or TPAB-diCF$_3$). Φ_{RB} is the $^1O_2$ quantum yield of RB, which is 0.75 in water.

Table S1. The calculations and data of the DFT analysis of the compounds.

Compounds	**TPAB-CF$_3$**	**TPAB-diCF$_3$**
LUMO	-0.08478	-0.09158
HOMO	-0.18276	-0.18482
ΔE_{H-L} [(LUMO-HOMO)*27.21]	2.666 eV	2.537 eV
S_1	2.1473	2.0482
T_1	1.9267	1.532
ΔE_{S1-T1} [S_1-T_1]	0.221 eV	0.195 eV

Table S2. Photophysical properties of lysosomes probes and their applications.

Lysosomes probes	$\lambda_{ex}/\lambda_{em}$, nm	Application
MTMM[2]<br>PTMM	420/570<br>421/571(THF/H_2O)	Lysosomes imaging on Cells and PDT
MP-TPEDCH[3]	352/654(DMF/H_2O)	Lysosomes imaging on Cells,PDT on cells and tumor-bearing mice
MPAA,MPAN,MPAT[4]	360/530,442/617<br>500/677(THF/H_2O)	Lysosomes imaging on Cells, Zebrafish and PDT
BODIPY-TPA[5]	563/682(THF/H_2O)	Lysosomes imaging on Cells and PDT on cells
TTRh-CN[6]	540/740(DMSO/H_2O)	Two-Photon Fluorescence Imaging in Cells and Tissue，PDT on cells and main organs of tumor-bearing mice

TPTDC[7]	511/758(THF/H_2O)	Two-Photon Fluorescence Imaging in Cells and Tissue，PDT on cells and main organs of tumor-bearing mice
BTZPP NPs[8]	450/635(THF/H_2O)	Imaging in Cells，PDT on cells and main organs of tumor-bearing mice
TPAB-CF_3 (this work)<br>TPAB-diCF_3 (this work)	343/577(DMSO/H_2O)<br>345/601	Lysosomes imaging and PDT on Cells

1. Wu, W.; Mao, D.; Xu, S.; Ji, S.; Hu, F.; Ding, D.; Kong, D.; Liu, B. High performance photosensitizers with aggregation-induced emission for image-guided photodynamic anticancer therapy. *Mater. Horiz.*, **2017**, *4*, 1110–1114.
2. Zhuang, J.B.; Yang, H.X.; Li, Y.; Wang, B.; Li, N.; Zhao, N. Efficient photosensitizers with aggregation-induced emission characteristics for lysosome- and Gram-positive bacteria-targeted photodynamic therapy. *Chem. Commun*. **2020**, *17*, 2630–2633.
3. Huang, W.D.; Zhang, Y.J.; Tan, X.Y.; Wang, N.; Wang, J.H.; He, M.L.; Peng, J.; Hu, J.; Zhao, Y.; Wang, S. An AIEgen-based photosensitizer for lysosome imaging and photodynamic therapy in tumor. *Sens. Actuators B Chem*. **2021**, *335*, 129698.
4. Dai, Y.P.; He, F.R.; Ji, H.F.; Zhao, X.X.; Misal, S.; Qi, Z.J. Dual-functional NIR AIEgens for high-fidelity imaging of lysosomes in cells and photodynamic therapy. *ACS Sens*. **2020**, *5*, 225–233.
5. Deng, J.R.; Yang, M.Q.; Li, C.; Liu, G.Y.; Sun, Q.; Luo, X.G.; Wu, F. Single molecular-based nanoparticles with aggregation-induced emission characteristics for fluorescence imaging and efficient cancer phototherapy. *Dyes Pigments* **2021**, *187*, 109130.
6. Zhou, Y.P.; Zhang, D.; He, G.H.; Liu, C.; Tu, Y.N.; Li, X.; Zhang, Q.; Wu, X.; Liu, R. A lysosomal targeted NIR photosensitizer for photodynamic therapy and two-photon fluorescence imaging. *J. Mater. Chem. B* **2021**, *9*, 1009.
7. Liao, Y.H.; Wang, R.L.; Wang, S.Z.; Xie, Y.F.; Chen, H.H.; Huang, R.J.; Shao, L.; Zhu, Q.; Liu, Y. Highly efficient multifunctional organic photosensitizer with Aggregation-Induced Emission for In vivo bioimaging and photodynamic therapy. *ACS Appl. Mater. Interfaces* **2021**, *13*, 54783–54793.
8. Huang, L.; Qing, D.Y.; Zhao, S.J.; Wu, X.L.; Yang, K.; Ren, X.J.; Zheng, X.; Lan, M.; Ye, J.; Zeng, L.; et al. Acceptor-donor-acceptor structured deep-red AIE photosensitizer: Lysosome-specific targeting, in vivo long-term imaging, and effective photodynamic therapy. *Chem. Eng. J.* **2022**, *430*, 132638.
